# Pre-admission Nutritional Risk as an Independent Predictor of 28-Day Mortality After Hip Fracture: A Retrospective Cohort Study

**DOI:** 10.7759/cureus.116040

**Published:** 2026-09-10

**Authors:** Gautham Sreekumar, Saurav Krishnan, Tharmesh Sivananthan, Aysha Rajeev

**Affiliations:** 1 Gastroenterology, Gateshead Health NHS Foundation Trust, Gateshead, GBR; 2 General Medicine, Northumbria Healthcare NHS Foundation Trust, Newcastle, GBR; 3 Pulmonology, Gateshead Health NHS Foundation Trust, Gateshead, GBR; 4 Trauma and Orthopaedics, Gateshead Health NHS Foundation Trust, Gateshead, GBR

**Keywords:** hip fracture, malnutrition, mortality, must score, nutritional screening, older adults, orthogeriatrics

## Abstract

Background: Hip fracture carries a substantial risk of early mortality in older adults. Malnutrition is common in this population and is closely correlated with age, frailty, and cognitive impairment, making its independent contribution to short-term mortality difficult to disentangle from these established prognostic factors. Whether nutritional risk on admission predicts 28-day mortality after accounting for age, sex, anesthetic risk grade, and cognitive status remains uncertain.

Methods: We conducted a retrospective cohort study of 709 consecutive patients admitted with hip fracture. Nutritional risk was assessed on admission using the Malnutrition Universal Screening Tool (MUST) and categorized as normal or any nutritional risk (at-risk and malnourished combined) for the primary analysis. The primary outcome was all-cause mortality within 28 days. Eighteen patients with an ungradable American Society of Anesthesiologists (ASA) grade were excluded, leaving 691 patients in the final analysis. Multivariable logistic regression estimated the association between nutritional risk and 28-day mortality, adjusting for age, sex, ASA grade, and admission cognitive status (Abbreviated Mental Test Score (AMTS)). Sensitivity analyses examined a three-category MUST classification; exploratory analyses examined post-admission delirium and mobilization status.

Results: Of 691 patients, 128 (18.5%) had nutritional risk on admission. Twenty-eight-day mortality was higher in this group than in patients with normal nutritional status (14.8% vs 5.2%; p < 0.001). Nutritional risk remained independently associated with mortality after adjustment (adjusted odds ratio (OR) 3.24, 95% confidence interval (CI) 1.65-6.35; p = 0.0006), with minimal change from the unadjusted estimate (OR 3.21, 95% CI 1.74-5.93), indicating the association is not substantially explained by age, sex, ASA grade, or cognition. Male sex (adjusted OR 3.31; p = 0.0003) and lower AMTS (adjusted OR 0.90 per point; p = 0.009) were independently predictive. In a sensitivity analysis using the three-category MUST classification, the "at-risk" group remained independently associated with mortality (adjusted OR 3.87, 95% CI 1.87-7.99), while the "malnourished" group did not reach statistical significance (adjusted OR 1.77, 95% CI 0.48-6.52), likely reflecting limited power in this smaller subgroup. Delirium severity and failure to mobilize postoperatively were both strongly associated with mortality in exploratory analyses (both p < 0.001) but were excluded from the primary model as post-admission factors.

Conclusion: Admission nutritional risk, as identified by the MUST, was associated with 28-day mortality following hip fracture, independent of age, sex, ASA grade, and cognitive status, although residual confounding by unmeasured factors cannot be excluded. These findings support the value of early nutritional assessment as part of a broader, multidimensional approach to identifying patients at increased risk of early death.

## Introduction

Hip fractures impose a substantial burden of loss of independence, morbidity, and mortality in older adults [1-3]. The reported 30-day mortality is between 5% and 10%. These numbers rise with comorbidities, frailty, and cognitive impairment at presentation [4,5]. Consequently, identifying readily available, modifiable, or early-measurable risk factors is important for prognostication and preoperative optimization.

Malnutrition is common in older adults with hip fracture, though prevalence estimates vary by population and assessment methods [6-9]. Poor nutrition is linked to impaired wound healing, infections, sarcopenia, reduced functional recovery, and prolonged hospitalization [7,8], making it a modifiable determinant of patient outcomes.

The Malnutrition Universal Screening Tool (MUST) is a simple, widely used instrument for the identification of nutritional risk on admission [10,11] and is therefore well suited for early risk stratification if its association with hard outcomes can be confirmed. A recent single-center UK study using MUST showed higher mortality among hip fracture patients who were at nutritional risk [9].

Although the association between malnutrition and adverse outcomes after hip fracture is well established, most prior studies have not adjusted for the principal covariates known to influence both nutritional status and mortality, limiting confidence that the reported associations reflect an independent effect rather than confounding by age, comorbidity, or cognitive impairment. MUST was selected as the exposure measure because it is validated, rapid to administer, and already recommended for routine use in UK clinical practice, and captures a composite of body mass index (BMI), recent weight loss, and acute illness effect rather than a single biochemical marker. Twenty-eight-day mortality was chosen as the primary outcome because it captures the early peri-operative period during which nutritional vulnerability is most likely to influence physiological reserve and complication risk, while remaining a clinically actionable and widely reported benchmark for comparison with existing hip fracture risk prediction tools.

However, documented correlations between malnutrition and death may partially represent residual confounding rather than an independent effect because age, sex, anesthetic risk, frailty, and cognitive impairment are independently linked to both nutritional status and mortality [1,4]. For nutritional risk to be used in clinical assessment, it is crucial to clarify if it provides prognostic information beyond these recognized standards.

The primary aim of this study was to determine the association between nutritional risk, assessed by MUST on admission, and 28-day mortality after hip fracture, adjusting for age, sex, American Society of Anesthesiologists (ASA) grade, and admission cognitive status. The secondary aim was to characterize the wider clinical circumstances by investigating univariate correlations between additional pre- and post-admission variables and 28-day mortality. This study was designed to determine whether nutritional risk provides prognostic information beyond established predictors of mortality after hip fracture.

## Materials and methods

Study design and setting

This retrospective cohort study used prospectively collected audit data from 709 consecutive patients admitted with hip fracture, comprising 60 demographic, pre-admission, perioperative, and outcome variables. Data were drawn from the hospital's National Hip Fracture Database (NHFD) audit dataset. Patients were admitted between January 4, 2022, and December 30, 2023. All patients underwent surgery within 48 hours of presentation, in line with national standards; time to surgery did not differ appreciably between patients with and without nutritional risk on admission.

Participants

Eligibility extended to all patients with a recorded hip fracture presentation. Eighteen patients (2.5%) with an ungradable ASA physical status grade ("U") [12] were excluded from the primary analysis. No other covariate included in the primary analysis had missing data. The final analytic sample therefore comprises 691 patients.

Exposure

The primary exposure was nutritional risk on admission, assessed by MUST and categorized as normal, at risk, or malnourished. MUST categorization was based on three components: BMI, unintentional weight loss, and the acute disease effect. Scores of 0, 1, and ≥2 were classified as normal, at risk, and malnourished, respectively [10]. For the primary analysis, the at-risk and malnourished categories were combined into a single nutritional risk group to maximize power and avoid instability from the small malnourished subgroup (n = 34). A secondary analysis retained the three-category classification to assess evidence of a graded association.

Outcome

The primary outcome was all-cause mortality within 28 days of admission, recorded as a binary outcome.

Covariates

Covariates were selected for established prognostic relevance and complete baseline data: age, sex, ASA grade (1-5), and admission cognitive status (Abbreviated Mental Test Score (AMTS); range 0-10). In the Nottingham Hip Fracture Score, AMTS is a validated independent predictor of mortality, supporting its inclusion here [5]. MUST, ASA grade, AMTS, and pre-fracture mobility were each assessed using distinct instruments, by different clinical teams, at different stages of admission, with no overlapping items - MUST is derived solely from BMI, weight loss, and acute disease effect. This measurement independence allowed each to be entered as a separate covariate in the multivariable model, so that the effect of nutritional risk could be estimated over and above any shared variance with these factors.

Delirium severity (4AT), administered postoperatively by the treating medical team, and postoperative mobility status were excluded from the primary analysis due to being mediators rather than confounders. Both variables were complete for all 691 patients in the analytic sample. "Failure to mobilize postoperatively" was recorded as "no," sub-categorized by reason (clinical contraindication, agitation or confusion, inadequate pain control, symptomatic hypotension, or resource limitation), by nursing or physiotherapy staff. These factors were examined separately in secondary analyses. Additional candidate confounders - serum creatinine, vitamin D status, fracture type, pathological fracture status, and bone protection therapy - were assessed univariately for a multivariable model where appropriate.

Statistical analysis

Baseline characteristics were compared between nutritional risk groups using chi-squared tests for categorical variables and Welch's t-test or ANOVA for continuous variables. The unadjusted association between nutritional risk and mortality was assessed by the chi-squared test, with odds ratios (ORs) and 95% confidence intervals (CIs) from standard 2 × 2 methods.

Multivariable logistic regression estimated the adjusted association between nutritional risk and mortality, controlling for age, sex, ASA grade, and AMTS. Candidate confounders were tested individually; a change in the nutritional risk OR of >10%, or a reduction in Akaike information criterion (AIC) of ≥2, was used as the criterion for retention, to preserve parsimony and sample size. Candidate selection combined univariable screening for association with mortality with clinical judgment, prioritizing variables with established prognostic relevance in hip fracture populations, to balance statistical evidence with domain knowledge rather than relying on statistical significance alone. A sensitivity analysis was conducted using the original three-category MUST classification (normal, at risk, and malnourished) to explore the presence of a dose-response relationship between nutritional status and mortality.

Secondary exploratory analyses examined the univariate associations between post-admission clinical factors, including 4AT delirium score, postoperative mobilization status, falls assessment, pressure ulcer development, and operative status, and 28-day mortality. These variables were not included in the primary adjusted model for the reasons described above.

All statistical tests were two-sided, and statistical significance was defined as p < 0.05. Effect estimates are presented as ORs with 95% CIs. Statistical analyses were performed using Python (Python Software Foundation, Wilmington, DE, USA), with independent verification of the primary and sensitivity models performed in R (R Foundation for Statistical Computing, Vienna, Austria). Test statistics (t for Welch's t-test, χ² for Pearson's chi-squared test, or Wald z for logistic regression coefficients, as applicable) are reported alongside p-values throughout the Results section.

## Results

Participants

Of 709 patients in the dataset, 18 (2.5%) were excluded due to an ungradable ASA grade, leaving 691 patients in the primary analysis. Of these, 128 (18.5%) had any nutrition risk on admission by MUST score (94 at risk, 34 malnourished), and 563 (81.5%) had normal nutrition status.

Baseline characteristics

Patients with nutrition risk on admission differed significantly from those with normal nutrition status across several baseline characteristics (Table 1). Patients with nutrition risk were more often female (78.9% vs. 66.6%, p = 0.009), had higher ASA grades (96.1% grade ≥ 3 vs. 83.8%, p < 0.001), had lower cognitive function on admission (mean AMTS 6.10 vs. 7.28, p = 0.002), and were less likely to be freely mobile pre-fracture (33.6% vs. 45.6%, p = 0.017). Age did not differ significantly between groups (83.2 vs. 82.6 years, p = 0.51).

**Table 1:** Baseline characteristics by nutrition risk status Test statistics: Welch's t-test for continuous variables (age, AMTS); Pearson's χ² test with Yates' continuity correction for categorical variables (sex, ASA grade, and mobility). ASA: American Society of Anesthesiologists; AMTS: Abbreviated Mental Test Score

Characteristic	Normal (n = 563)	Any nutritional risk (n = 128)	Test statistic	p
Age (years), mean (SD)	82.6 (8.8)	83.2 (9.1)	t(185.3) = -0.66	0.51
Female sex, n (%)	375 (66.6)	101 (78.9)	χ²(1) = 6.80	0.009
ASA grade ≥ 3, n (%)	472 (83.6)	123 (96.1)	χ²(1) = 12.09	<0.001
AMTS, mean (SD)	7.28 (3.53)	6.10 (3.92)	t(176.8) = 3.12	0.002
Freely mobile pre-fracture, n (%)	257 (45.6)	43 (33.6)	χ²(1) = 5.69	0.017

Primary outcome

Twenty-eight-day mortality occurred in 19 of 128 patients (14.8%) with nutrition risk, compared with 29 of 563 patients (5.2%) with normal nutrition status (χ²(1) = 13.70, p < 0.001; Figure 1, Table 2). Nutritional risk was associated with significantly higher unadjusted odds of mortality (OR 3.21, 95% CI 1.74-5.93). After adjustment for age, sex, ASA grade, and AMTS, the association remained strong (adjusted OR 3.24, 95% CI 1.65-6.35, p = 0.0006); the minimal change from the unadjusted estimate suggests it is not substantially explained by these baseline differences. In the fully adjusted model, male sex (adjusted OR 3.31, 95% CI 1.72-6.36, p = 0.0003) and lower AMTS (adjusted OR 0.90 per point, 95% CI 0.82-0.97, p = 0.009) were also independently associated with 28-day mortality. Age and ASA grade were not independently significant after adjustment (p = 0.12 and p = 0.11, respectively; Figure 2).

**Figure 1:**
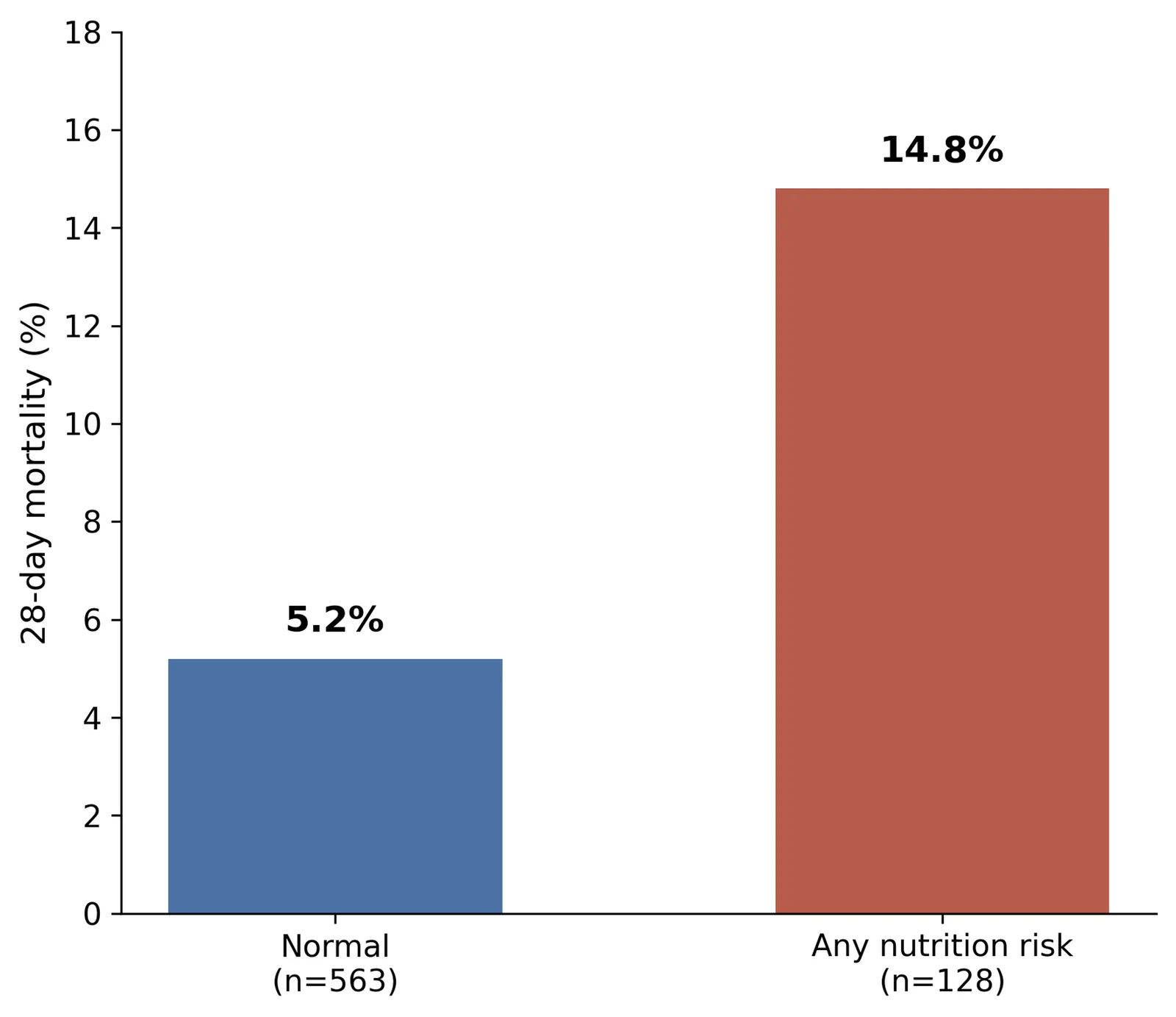
Twenty-eight-day mortality rate by nutrition risk status (any risk vs. normal)

**Table 2:** Unadjusted and adjusted association between nutrition risk and 28-day mortality *Adjusted for age, sex, ASA grade, and AMTS. Wald z-statistics are reported for each adjusted odds ratio (OR) from the multivariable logistic regression model. ASA: American Society of Anesthesiologists; AMTS: Abbreviated Mental Test Score; CI: confidence interval

Variable	Unadjusted OR (95% CI)	Adjusted OR (95% CI)*	Wald z	p (adjusted)
Nutrition risk present (vs. normal)	3.21 (1.74-5.93)	3.24 (1.65-6.35)	3.43	0.0006
Male sex	-	3.31 (1.72-6.36)	3.59	0.0003
AMTS (per point)	-	0.90 (0.82-0.97)	-2.62	0.009
Age (per year)	-	1.03 (0.99-1.07)	1.54	0.12
ASA grade (per point)	-	1.58 (0.90-2.75)	1.58	0.11

**Figure 2:**
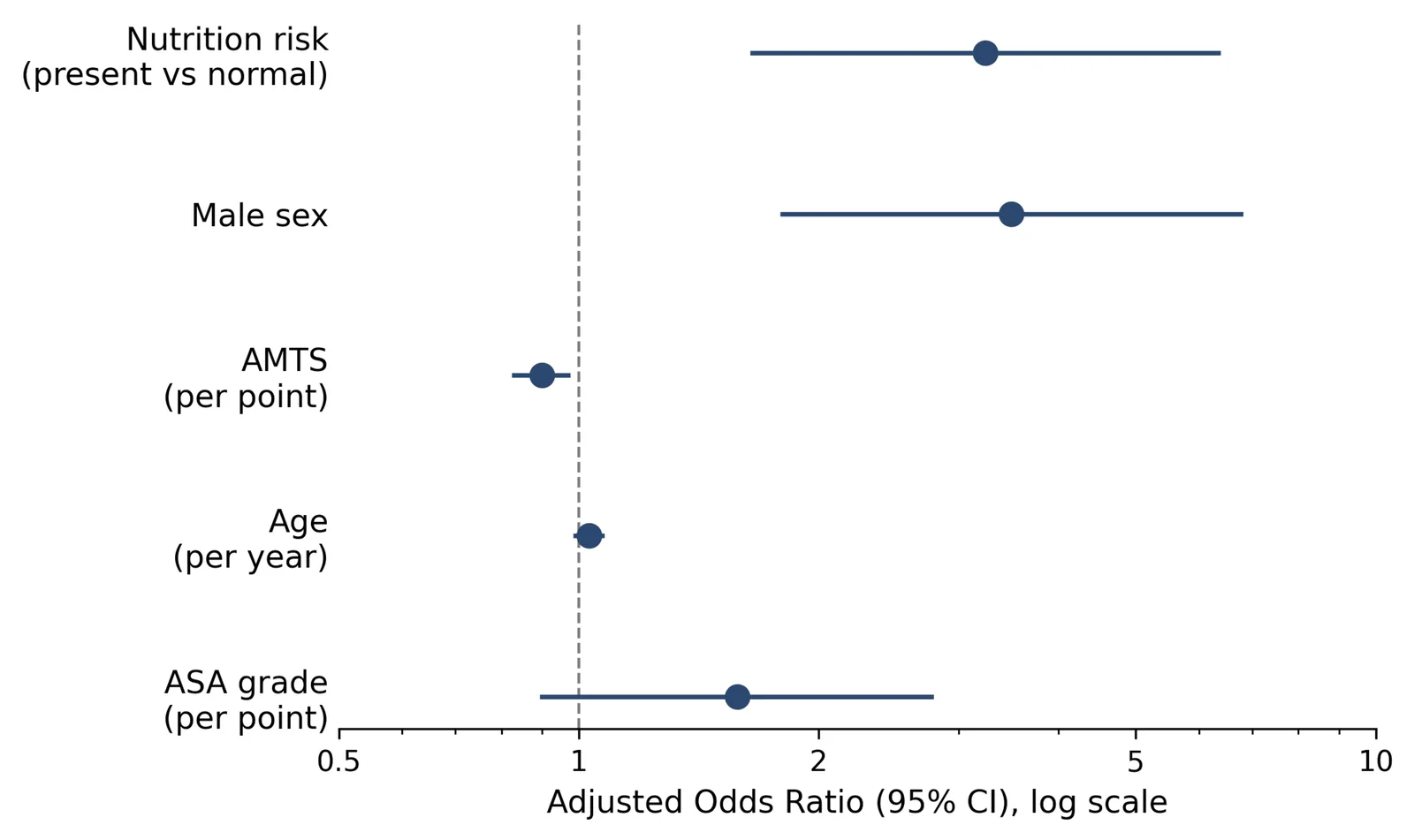
Forest plot of adjusted odds ratios (ORs) (95% CI) for 28-day mortality. Dashed line at OR = 1 indicates no effect ASA: American Society of Anesthesiologists; AMTS: Abbreviated Mental Test Score; CI: confidence interval

Sensitivity analysis: three-category nutrition risk

Using the original three-category exposure, 28-day mortality occurred in 29/563 (5.2%) patients with normal nutrition (Figure 3), 16/94 (17.0%) of those at risk, and 3/34 (8.8%) of those classified as malnourished. In the adjusted model, "at risk" status remained strongly and independently associated with mortality (adjusted OR 3.87, 95% CI 1.87-7.99, z = 3.66, p < 0.001), while the "malnourished" category did not reach statistical significance (adjusted OR 1.77, 95% CI 0.48-6.52, z = 0.85, p = 0.39), likely reflecting limited statistical power in this small subgroup (n = 34, three events) rather than a true absence of effect.

**Figure 3:**
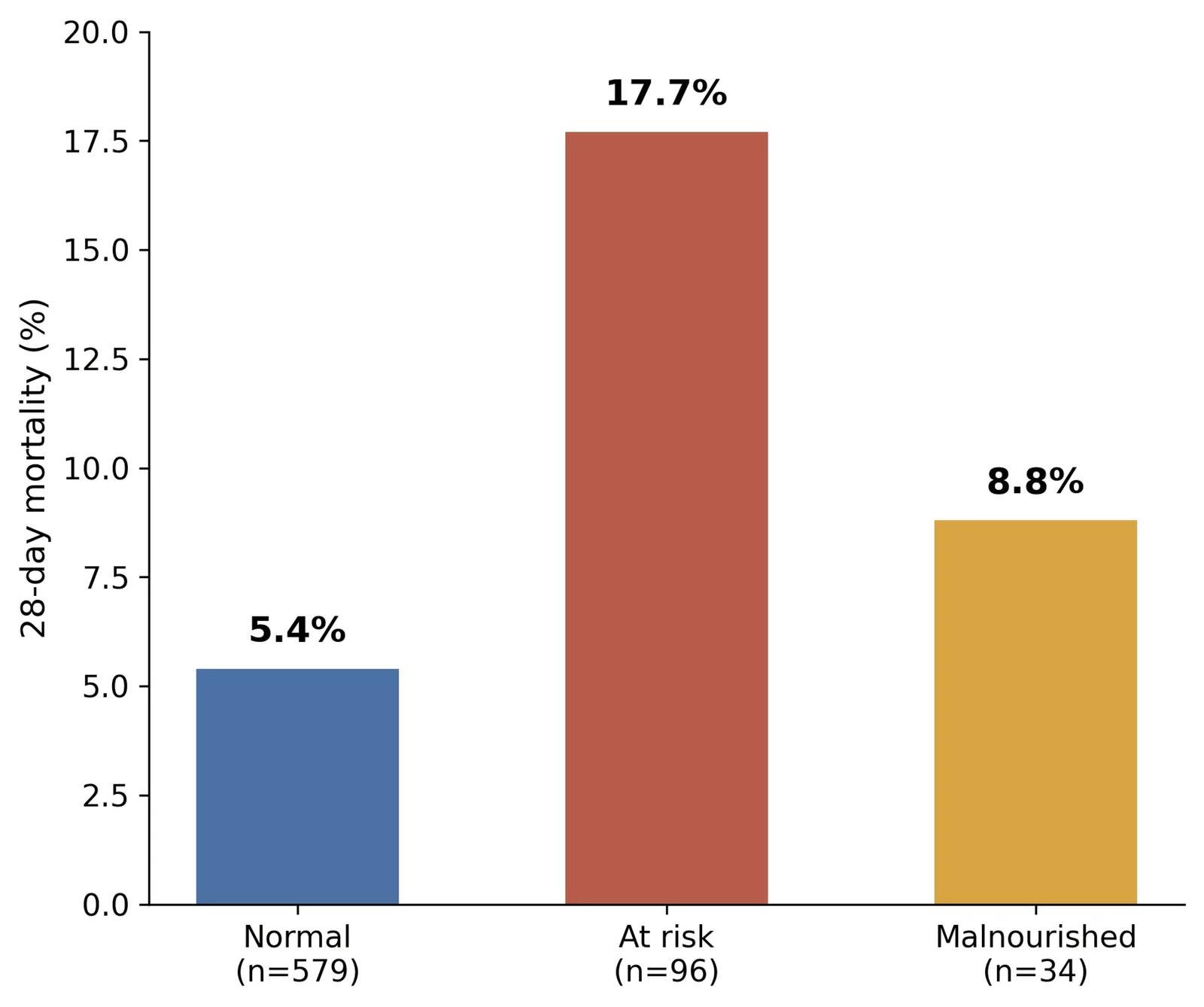
Twenty-eight-day mortality rate by three-category MUST classification (normal/at risk/malnourished) MUST: Malnutrition Universal Screening Tool

Additional confounder assessment

Vitamin D status was categorized as severe deficiency (<25 nmol/L), deficiency (25-<50 nmol/L), insufficiency (50-75 nmol/L), or sufficient (>75 nmol/L). Twenty-eight-day mortality was 9.5% (21/220) in the severely deficient group, 6.2% (11/176) in the deficient group, 1.9% (3/161) in the insufficient group, and 8.3% (9/108) in the sufficient group. The vitamin D category was significantly associated with mortality univariately (χ²(3) = 9.50, p = 0.023) but showed no consistent dose-response pattern, and its inclusion did not meaningfully alter the nutritional risk estimate (adjusted OR 2.99, 95% CI 1.44-6.19, p = 0.003). Fracture location, fracture type, pathological fracture status, fracture side, and serum calcium were not significantly associated with mortality (all p > 0.05) and were not retained in the final model.

Secondary exploratory analysis: post-admission factor

Higher delirium severity (4AT mean 4.62 in those who died vs. 2.07 in survivors, t(50.4) = 4.12, p < 0.001) and failure to mobilize postoperatively (14.6% vs. 4.9% mortality, χ²(1) = 14.04, p < 0.001) were both strongly associated with 28-day mortality but excluded from the primary model as post-admission factors. Falls assessment, pressure ulcer occurrence, and postoperative weight-bearing status showed no association, largely reflecting limited variability in this cohort.

## Discussion

In this cohort of 691 patients admitted with hip fracture, nutritional risk identified on admission using the MUST was independently associated with more than a threefold increase in 28-day mortality after adjustment for age, sex, ASA grade, and admission cognitive function. Importantly, the adjusted OR (3.24) was almost identical to the unadjusted estimate (3.21), indicating minimal confounding by these established prognostic variables. This suggests that nutritional risk captures an important dimension of physiological vulnerability that is not adequately explained by chronological age, comorbidity, or cognitive impairment alone.

These findings are consistent with an extensive body of literature demonstrating that malnutrition adversely influences outcomes following hip fracture. Previous UK studies using MUST have similarly reported substantially higher mortality among patients identified as nutritionally at risk [9], while systematic reviews consistently demonstrate two- to threefold increases in mortality together with higher rates of postoperative complications, prolonged hospitalization, impaired functional recovery, and increased institutionalization among malnourished patients [6-8]. The magnitude of association observed in the present study lies comfortably within this reported range, supporting the external validity of our findings despite differences in patient populations and healthcare settings.

The relationship between malnutrition and mortality is biologically plausible. Malnutrition contributes to sarcopenia, frailty, impaired immune competence, delayed wound healing, chronic inflammation, and diminished cardiopulmonary reserve [7]. Collectively, these factors reduce an individual's ability to tolerate the physiological stress associated with fracture, surgery, anesthesia, and the postoperative inflammatory response [13]. Furthermore, inadequate nutritional reserves may impair rehabilitation by limiting muscle protein synthesis and functional recovery, increasing susceptibility to complications such as infection, pressure ulcers, and prolonged immobility [14]. Rather than representing a simple marker of ill health, nutritional risk may therefore directly contribute to poorer clinical outcomes through several interrelated mechanisms.

The observation that the adjusted association remained largely unchanged after controlling for age and ASA grade is noteworthy. Age and comorbidity are among the strongest predictors incorporated into established risk prediction models, including the Nottingham Hip Fracture Score, yet nutritional status appears to provide prognostic information beyond these conventional variables [13,14]. This suggests that routine nutritional screening may identify high-risk patients who would not necessarily be recognized through existing risk stratification tools alone [9]. Given that MUST is inexpensive, rapid to administer, and already recommended within UK clinical practice, these findings support its continued routine use at hospital admission and highlight its potential value in early prognostic assessment.

Male sex was independently associated with increased 28-day mortality, consistent with previous studies reporting disproportionately poorer outcomes in men following hip fracture despite their lower incidence of fracture [15]. Several mechanisms have been proposed, including greater pre-fracture comorbidity, higher rates of postoperative infection, delayed presentation, and reduced physiological reserve [16]. Although the present study was not designed to investigate sex-specific mechanisms, our findings reinforce evidence that male sex should remain an important consideration during early risk assessment.

Admission cognitive function measured using the AMTS also independently predicted mortality [5]. Cognitive impairment is well recognized as a marker of frailty and vulnerability and forms a component of several validated prognostic models [17]. Reduced cognition may increase mortality through multiple pathways, including impaired communication, reduced participation in rehabilitation, increased risk of delirium, aspiration, and poorer adherence to postoperative care [17,18]. The independent contribution of AMTS in our model supports its continued inclusion in prognostic assessment alongside nutritional screening.

Although age and ASA grade were significant in univariable analyses, neither remained independently associated with mortality after multivariable adjustment [19]. This most likely reflects shared variance between these variables and nutritional status, cognition, and sex, rather than suggesting that age or comorbidity are clinically unimportant. Older patients and those with greater comorbidity are more likely to be frail, cognitively impaired, and nutritionally vulnerable, resulting in attenuation of their independent statistical effects within the adjusted model [19]. Similar findings have been reported in multivariable studies examining overlapping domains of frailty in hip fracture populations [20].

These findings should be interpreted as evidence of an association that is independent of the specific covariates adjusted for in this analysis - age, sex, ASA grade, and cognitive status - rather than proof that nutritional risk is independent of all plausible confounders. Several domains of physiological vulnerability relevant to hip fracture outcomes were not comprehensively captured in this dataset, including frailty, polypharmacy, comorbidity burden (e.g., diabetes, renal impairment, and cardiac disease), biochemical markers of nutritional reserve such as serum albumin, and sarcopenia or broader bone health status. Residual confounding by these unmeasured factors cannot be excluded, and our findings are best understood as demonstrating a robust statistical association rather than proof of incremental predictive utility over existing risk stratification tools; formally establishing the latter would require comparing the discrimination and calibration of models with and without MUST, which was outside the scope of this study.

Analysis of MUST categories demonstrated that patients classified as "at risk" had the strongest association with mortality, whereas the "malnourished" category did not reach statistical significance - indeed, mortality was numerically higher in the "at risk" group than in the "malnourished" group, so these data do not demonstrate a clear, graded dose-response relationship between worsening nutritional status and mortality. This finding should be interpreted cautiously and does not necessarily represent a true reversal of effect. Only 34 patients were classified as malnourished, among whom three deaths occurred, resulting in limited statistical power and wide CIs. The absence of statistical significance is therefore unlikely to represent evidence of no association. Several alternative explanations may also contribute. It is possible that patients identified as malnourished received more intensive dietary and multidisciplinary nutritional support during admission, which may have attenuated their observed mortality relative to what would otherwise be expected; this hypothesis cannot be tested with the available data. The malnourished group may also differ systematically from the at-risk group in unmeasured baseline characteristics, such as pre-existing frailty, care home residence, or comorbidity burden, representing a heterogeneous subgroup with differing baseline characteristics or treatment pathways. Selection bias is a further possibility: the most severely malnourished individuals may have been more likely to die, or to be deemed unfit for surgery, before formal MUST assessment or study inclusion, which could paradoxically lower the observed mortality rate in the surviving malnourished cohort. Larger multicenter cohorts or pooled analyses will be required to determine whether mortality increases progressively with worsening nutritional status or whether the relationship becomes non-linear, or reverses, at the highest levels of nutritional deficit; this three-category analysis should be regarded as hypothesis-generating rather than confirmatory.

Vitamin D status demonstrated an association with mortality in univariate analysis but provided little additional prognostic value once nutritional risk and other covariates were included in the multivariable model [10,11]. Furthermore, no consistent dose-response relationship was observed across vitamin D categories. These findings suggest that while vitamin D deficiency and malnutrition frequently coexist, they are not interchangeable markers of physiological risk. Vitamin D may reflect one component of nutritional health or frailty, whereas MUST captures a broader multidimensional assessment incorporating BMI, recent weight loss, and the effects of acute illness [10,11]. Consequently, nutritional risk identified by MUST appears to provide more comprehensive prognostic information than vitamin D status alone [10,11].

Delirium severity and failure to mobilize postoperatively were both strongly associated with mortality but were intentionally excluded from the primary regression model because they occur after admission and may represent mediators rather than confounders of the relationship between nutritional status and mortality [13,14]. Malnutrition could plausibly increase susceptibility to delirium through metabolic vulnerability, impaired cerebral reserve, and inflammatory mechanisms, while simultaneously limiting the capacity for early mobilization through reduced muscle strength and functional reserve [21]. Both postoperative delirium and delayed mobilization are independently associated with substantially increased mortality in hip fracture populations [21,22]. Consequently, adjusting for these variables may have obscured part of the effect of nutritional risk by controlling for factors lying on the causal pathway. Notably, both delirium and failure to mobilize are potentially modifiable or preventable through targeted perioperative interventions; even if part of the nutritional risk effect operates through these pathways, this does not diminish their relevance as actionable clinical targets for reducing mortality. Future studies incorporating formal mediation analysis could help quantify the proportion of excess mortality attributable to these intermediate mechanisms.

The findings have important clinical implications. Nutritional screening is already recommended for all hospital admissions, yet implementation and subsequent nutritional intervention remain variable in routine orthopedic practice. Identification of nutritional risk at admission provides an opportunity to initiate early dietetic assessment, optimize protein and energy intake, address micronutrient deficiencies, and implement multidisciplinary interventions that may improve postoperative recovery [23]. Although evidence that nutritional supplementation directly reduces mortality remains mixed, several randomized trials and systematic reviews have demonstrated reductions in complications, improved nutritional status, and enhanced functional recovery [24]. Our results strengthen the rationale for incorporating nutritional optimization into comprehensive ortho-geriatric care pathways and suggest that nutritional status should be considered when stratifying mortality risk after hip fracture.

Limitations

This study had few limitations. The retrospective, single-center design may limit generalizability and cannot exclude residual confounding from unmeasured factors such as frailty, polypharmacy, and comorbidity burden not captured by ASA grade alone; a formal frailty assessment was not performed. Data on nutritional interventions during admission and on cause of death were not available, precluding assessment of whether dietary support modified mortality risk or whether deaths were nutrition-related. The malnourished subgroup was small (n = 34), limiting precision and likely contributing to the counterintuitive finding that the "at risk" group showed a stronger mortality association than the "malnourished" group; this three-category analysis should be regarded as hypothesis-generating rather than confirmatory. Serum calcium and vitamin D status had incomplete data, and "failure to mobilize postoperatively" was not tied to a specific postoperative day, limiting precision for these variables. The incremental predictive value of MUST beyond existing clinical risk factors was not formally assessed, and would require comparison of model discrimination and calibration. Finally, as with all observational studies, causal inference cannot be established, and external validation in independent, multicenter cohorts is required.

## Conclusions

Nutritional risk identified by MUST on admission was associated with a more than threefold increase in 28-day mortality after hip fracture, independent of age, sex, ASA grade, and cognitive status, although residual confounding by unmeasured factors such as frailty and comorbidity burden cannot be excluded. These findings support the value of routine nutritional screening on admission as part of a broader, multidimensional assessment of risk after hip fracture. Prospective, multicenter studies are warranted to externally validate this association and to determine whether targeted nutritional interventions can improve outcomes in this high-risk population.
